# *A hypothetical model*: Chromatin remodelers couple with acetyltransferases to trigger the elongation of RNA polymerase II (pol II)

**DOI:** 10.3389/freae.2024.1439973

**Published:** 2024-07-31

**Authors:** Gongyi Zhang

**Affiliations:** 1 Department of Immunology and Genomic Medicine, National Jewish Health, Denver, CO, United States,; 2 Department of Immunology and Microbiology, School of Medicine, Anschutz Medical Center, Aurora, CO, United States

**Keywords:** SWI/SNF, RSC, INO80, SWR1, CHD1/3/4, ISW1b, SAGA, NuA4

## Abstract

Transcription is one of the central dogmas of life. Most genes in eukaryotes are transcribed by RNA polymerase II (Pol II). For Pol II to transcribe along the gene body, it must overcome nucleosomes, which are barriers for Pol II. It is still a mystery how Pol II ejects nucleosomes during transcription elongation. I hypothesize that a group of chromatin remodelers (SWI/SNF, RSC, SWR1, INO80) carry a group of histone acetyltransferases (NuA4, Spt-Ada-Gcn-acetyltransferase, NuA3) to deposit acetyl-groups on histone tails to generate pan-acetylated nucleosomes or fragile nucleosomes along gene bodies for Pol II to transcribe. Specifically, for the first round of transcription, the RSC complex works with NuA4 to acetylate histone tails of H2A and H4; the SWI/SNF complex carries SAGA to add acetyl-groups to histone tails of H2B and H3. For the second and subsequent rounds, SWR1 pairs with a piccolo NuA4 to acetylate the histone tails of H2A and H4 of newly inserted nucleosomes, while INO80 pairs with NuA3 to acetylate the histone tails of H2B and H3 within newly inserted nucleosomes along the gene body. After the mission is accomplished, ISW1b couples with Rpd3s to remove acetyl groups on H2A and H4, while CHD1 carries HDA1 along the gene body to remove acetyl groups on H2B and H3.

## Introduction

It has been nearly 80 years since DNA was first discovered to contain the genetic information of life (Avery et al., 1944). These genetic codes on DNA were transcribed into RNAs by RNA polymerases (RNAPs) (Roeder and Rutter, 1969; Hurwitz, 2005). The mechanism of transcription in simple organisms such as bacteria has been well characterized by a recruitment mechanism (Ptashne and Gann, 1997). The function and structure of RNAPs from bacteria, archaea bacteria, simple eukaryotes, and animals are highly conserved (Zhang et al., 1999; Cramer et al., 2000; Bernecky et al., 2016). RNAPs derived from bacteria or even from viruses can transcribe any DNA from either lower life or humans. However, due to the joining of nucleosomes, which is the basic unit of chromosomes in eukaryotes, RNAPs alone in eukaryotes cannot transcribe on DNAs associated with nucleosomes, which not only prohibits RNAP from binding to DNAs but also prevents their elongation along the gene body due to the tight association between DNA and histone octamers (Olins and Olins, 1974). Despite more than 50 years of effort, the interpretation of how RNAPs overcome nucleosomes during transcription in eukaryotes remains a mystery.

**FIGURE 1:**
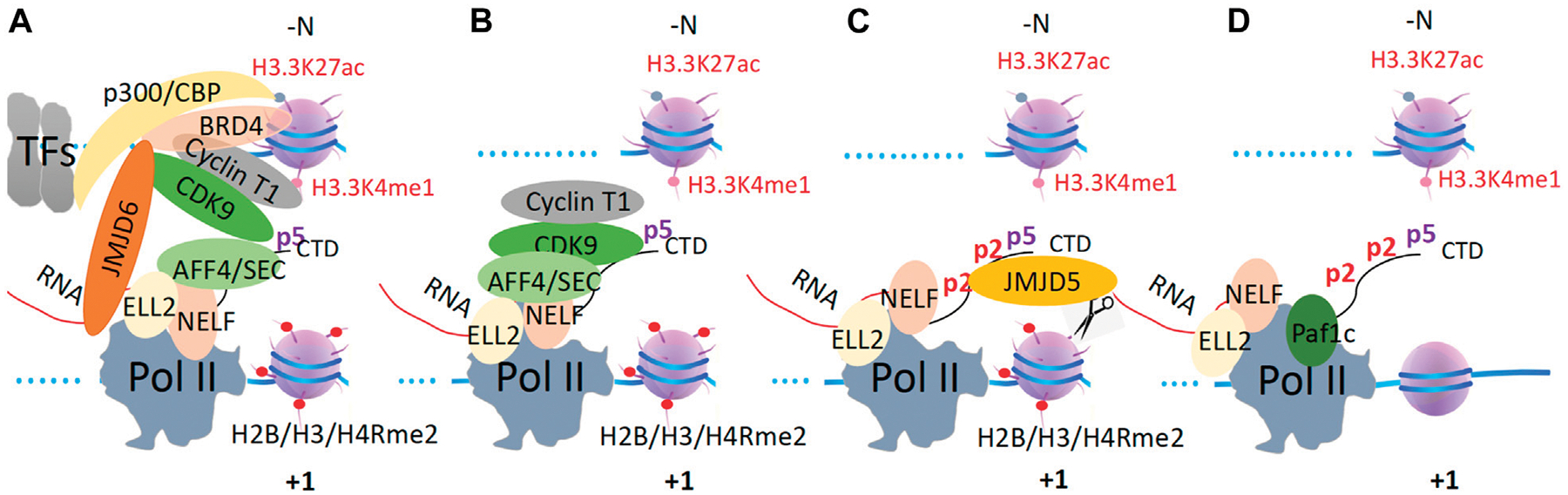
The promoter-proximal Pol II pausing regulation mechanism in metazoans. **(A)**. TFs work together to recruit p300/CBP to generate acetyl groups on nucleosomes at promoter/the enhancer, which recruits BRD4. BRD4 recruits CDK9 to SEC and Pol II with the help of JMJD6. **(B)**. ELL2 and NELF recruit SEC with CDK9 onto Pol II, which phosphorylates the CTD of Pol II. **(C)**. Ser2p-CDK9-CTD recruits JMJD5 to cleave arginine-methylated histone tails at the +1 nucleosome. **(D)**. Pol II overcomes the tailless +1 nucleosome to release paused Pol II and eject NELF and ELL2, which are replaced by Paf1c. Pafic will recruit other elongation factors to help Pol II overcome other nucleosomes along the gene body.

**FIGURE 2:**
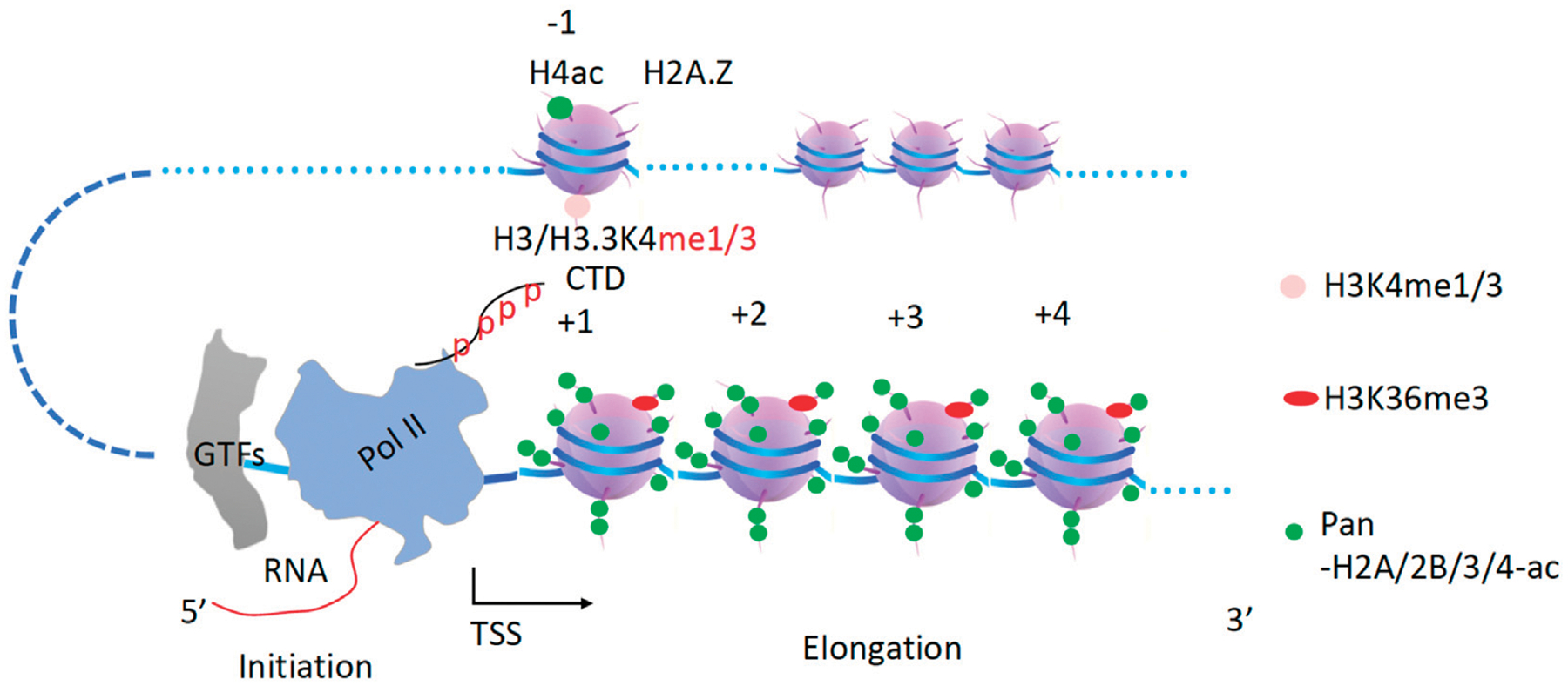
A model of how Pol II can transcribe along pan-acetylated nucleosomes *in vivo*. It is proposed that all nucleosomes within the gene body must be pan-acetylated at their tails for Pol II to be able to overcome them in the first round of transcription. CTD, C-terminal domain of Pol II. GTFs, general transcription factors. TSS, transcription start site.

In archaea, RNAPs were found to transcribe along the gene body without the need for any additional help. Interestingly, archaeal chromosomes also contain nucleosomes but lack histone tails (White and Bell, 2002; Koster et al., 2015). *In vitro* experiments have shown that RNA polymerase II (Pol II) also can transcribe along artificial nucleosomes without histone tails or histones with mimicked fully acetylated tails (Protacio et al., 2000), the conclusions have been further verified by single-molecule assays *in vitro* (Bintu et al., 2012). Our recent work has shown that tailless nucleosomes do exist in higher eukaryotic cells (Liu et al., 2017; Liu et al., 2018; Liu, Ramachandran, et al., 2020). The phenomenon of promoter-proximal Pol II pausing, which controls the expression of ~30–60% of genes and only occurs in metazoans (Jonkers and Lis, 2015), could be caused by the barrier of arginine-methylated nucleosomes at +1 from the transcription start sites (TSS) (Bondarenko et al., 2006; Mavrich et al., 2008; Weber et al., 2014; Voong et al., 2016; Liu et al., 2022). After decades of effort, my group showed that a subset of the Jumonji protein family, JMJD5 and JMJD7, can cleave histone tails with methylated arginines to generate “tailless nucleosome” through dual endo- and exopeptidase activities in metazoans (Liu et al., 2017; Liu et al., 2018). Detailed characterizations revealed that JMJD5 can specifically act on arginine methylated +1 nucleosomes of a large set of genes (Liu et al., 2020b). Furthermore, we found that there is a CTD (C-terminal domain) of the Pol II interaction domain (CID) within the N-terminal of JMJD5, which recognizes a unique phosphorylation pattern of CTD of Pol II (Liu et al., 2020b). More surprisingly, we found that this unique phosphorylation pattern of CTD of the Pol II is generated by CDK9 (Liu et al., 2020). We concluded that JMJD5 couples with CDK9 to release the paused Pol II into the active transcription elongation (Liu et al., 2020b). Another interesting discovery from our group showed that JMJD6 also acts as a dual-peptidase to cleave arginine methylated MeCPE protein within the 7SK snRNP complex so as to release CDK9 from the 7SK snRNP complex and recruited to Pol II by BRD4 (Hong et al., 2010; Lee et al., 2020). We concluded that the promoter-proximal Pol II pausing regulation, arginine methylation of histone tails at +1 nucleosomes, the cleavage of arginine-methylated histone tails by JMJD5/7, the high turnover rate of histones in non-proliferating cells, the tight control of CDK9 activity, the unique pattern of phosphorylation of the C-terminal domain of Pol II (CTD) generated by CDK9, the recruitment of CDK9 to Pol II by BRD4 and JMJD6, and possibly the p300/CBP as well as the participation of enhancers, could be coupled (Liu et al., 2020a; Liu et al., 2020b). Based on our novel findings and accumulating data from others, a simplified model of the promoter-proximal Pol II pausing regulation in metazoans could be derived (Figure 1). An emerging question that arises after solving the mechanism of Pol II pausing regulation in metazoans is how Pol II overcomes the resistance of the rest of the nucleosomes at +2, +3,…after the release from +1 in metazoans and all nucleosomes of housekeeping genes or all genes in simple eukaryotes, such as yeast. It has been found that transcriptional activation is coupled to the widespread acetylation of histone tails throughout the genome (Allis et al., 1980; Jeppesen and Turner, 1993). The enzymes responsible for the activities of acetylation of histone tails are well characterized (Brownell and Allis, 1995; Brownell et al., 1996; Smith et al., 1998; Allard et al., 1999; Clarke et al., 1999; John et al., 2000). Combined with the *in vitro* assays that Pol II can overcome nucleosomes with fully mimic acetylated histone tails (Protacio et al., 2000; Bintu et al., 2012), I attempt to speculate that there must be fully acetylated nucleosomes along gene bodies for Pol II to transcribe during the elongation process *in vivo* (Figure 2). However, how these acetyltransferases deposit acetyl groups on the histone tails along the gene body to promote Pol II elongation remains a mystery. In this essay, I will propose a hypothesis with a large group of evidence available to resolve this puzzle. Due to the lack of the C-terminal domain (CTD) for both RNA Polymerase I (Pol I) and RNA Polymerase III (Pol III), it is not clear whether pan-acetylation is also required for the activities of Pol I or Pol III. Other forms of transcription regulation mechanisms may likely help Pol I or Pol III to overcome the resistance of nucleosomes, which is beyond the scope of this essay.

## The hypothesis

In recent decades, a subset of the DEAD/H-box superfamily of RNA and DNA helicases, the SF2 family of DNA helicases (Singleton et al., 2007), has been shown to be essential for transcriptional activation in eukaryotes (Varga-Weisz, 2001). Although the primary function of all helicases by definition is to break the hydrogen bonds within the double-stranded DNAs (dsDNAs) or RNAs (dsRNAs), while the translocation property (sliding) driven by hydrolysis of ATP along the dsDNA (Velankar et al., 1999) and dsRNAs (Sinha et al., 2018) has not been well appreciated. This property may also apply to the function of the MCM complex (the minichromosome maintenance protein complex), which has been found to bind to dsDNA *in vitro* (Li et al., 2015; Noguchi et al., 2017). The Remodels Structure of Chromatin (RSC) complex has been reported to translocate DNA along the nucleosomes (Saha et al., 2002), while Imitation SWI (ISWI, Imitation SWI/SNF) has been found to translocate along the helical DNA (Whitehouse et al., 2003). Overall, a large group of chromatin remodelers translocate along nucleosome-constructed chromosomes (Gkikopoulos et al., 2011; Yen et al., 2012; Narlikar et al., 2013).

**FIGURE 3:**
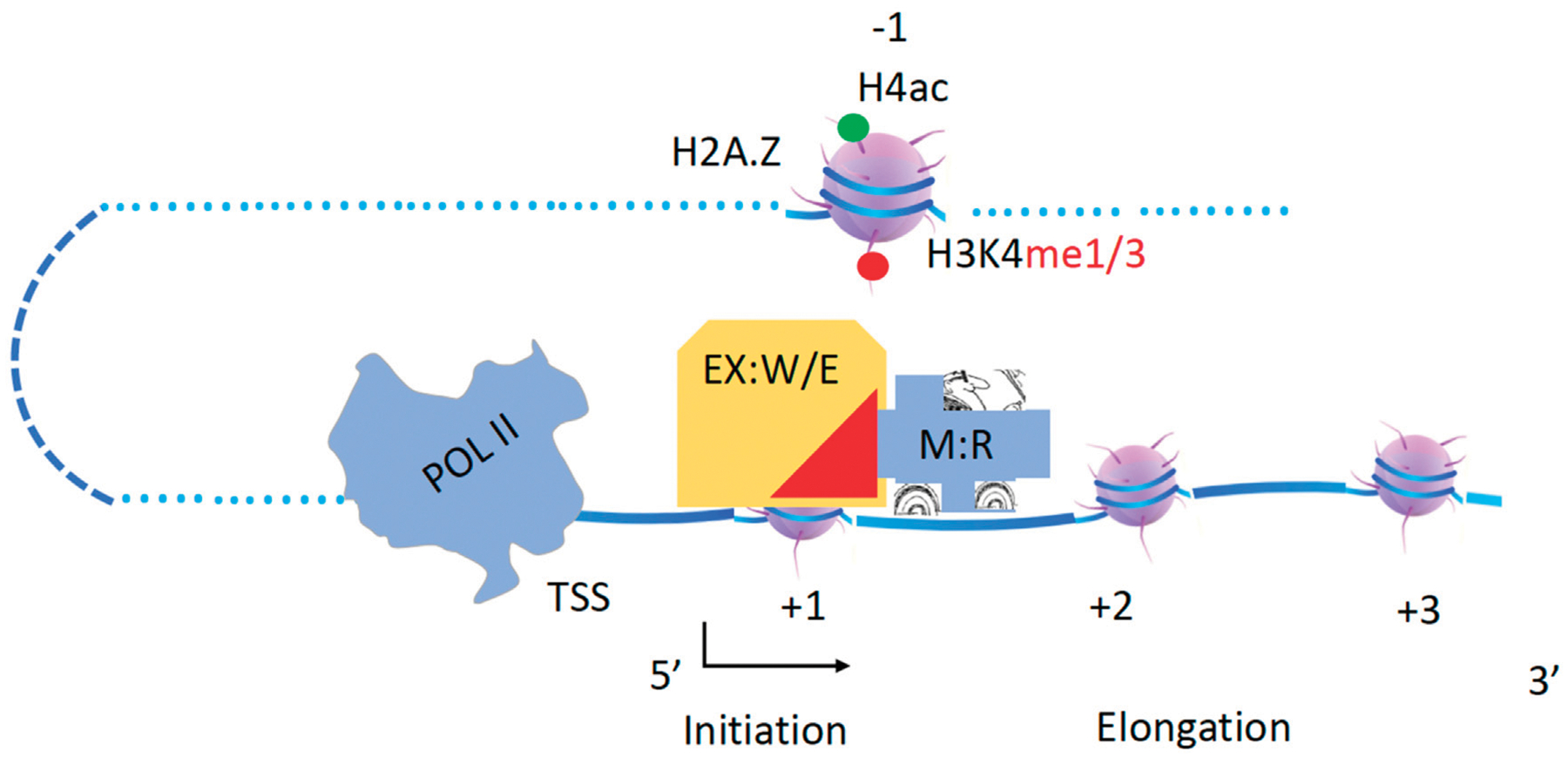
A hypothesis of chromatin remodelers as drivers to carry histone modification complexes to carry out modifications along gene bodies. Executives(EX): Writers(W) and Erasers(E); Engine or Motor(M): Remodelers (R).

The helicase within the Dicer complex carries the RNase III enzyme to generate microRNAs (miRNAs) and small interfering RNAs (siRNAs) along dsRNAs (Sinha et al., 2018). Following a similar mode, the Mi-2/NuRD (nucleosome remodeling deacetylase) complex contains a remodeler (CHD3/4) and a deacetylase in the higher eukaryotes working along the gene body (Tong et al., 1998; Xue et al., 1998; Zhang et al., 1998a). This complex is conserved in eukaryotes, Mit1 (CHD1 in budding yeast, CHD3/4 in animals) in the SHREC complex (Snf2/Hdac Repressive Complex, NuRD complex in animals) couples with the histone deacetylase Clr3 (HDA1 in budding yeast, HDAC III in animals) to remove acetyl groups from histone tails (Job et al., 2016). Isw2, a component of ISWI, and the Rpd3 complex (HDAC I/II) were pulled down by a repressor, ume6, to work together to repress gene expression of a group of genes, although it is not verified whether they are associated with each other (Goldmark et al., 2000), while ISWI was shown to interact with Rpd3 later (Burgio et al., 2008). It has been well characterized that SWR1 associates with a form of NuA4 through Yaf9 to carry out the enzymatic activities of NuA4 (Keogh et al., 2006; Altaf et al., 2010; Wang et al., 2018). Recently, a high-resolution single-molecule tracking reported that CHD1 carries the FACT complex along the gene bodies in yeast (Jeronimo et al., 2021). Based on these accumulating data, I hypothesize that acetyltransferase complexes including SAGA, NuA4 (two forms), and NuA3, and deacetylase complexes including Rpd3 and HDA1 must be coupled to one of the chromatin remodelers, CHD1, RSC, SWI/SNF, INO80, SWR1, ISWI, or CHD3/4, to carry out their specific enzymatic function along the gene body (Figure 3). In this model, histone acetyltransferases (W, writers) and acetyl deacetylases (E, erasers) act as executive cargoes (EX) while Chromatin remodelers (R) act as drivers or motors (M). This principle should also apply to other enzymes that modify histone tails along the gene body, such as Rad6/Bre1, Dot1, Set2, jh2, etc. In the following sections, I will try to pair them up based on published evidence.

## CHD1 drives Rad6/bre1 and Dot1 to ubiquitinate H2B and methylate H3K79 along gene bodies

**FIGURE 4:**
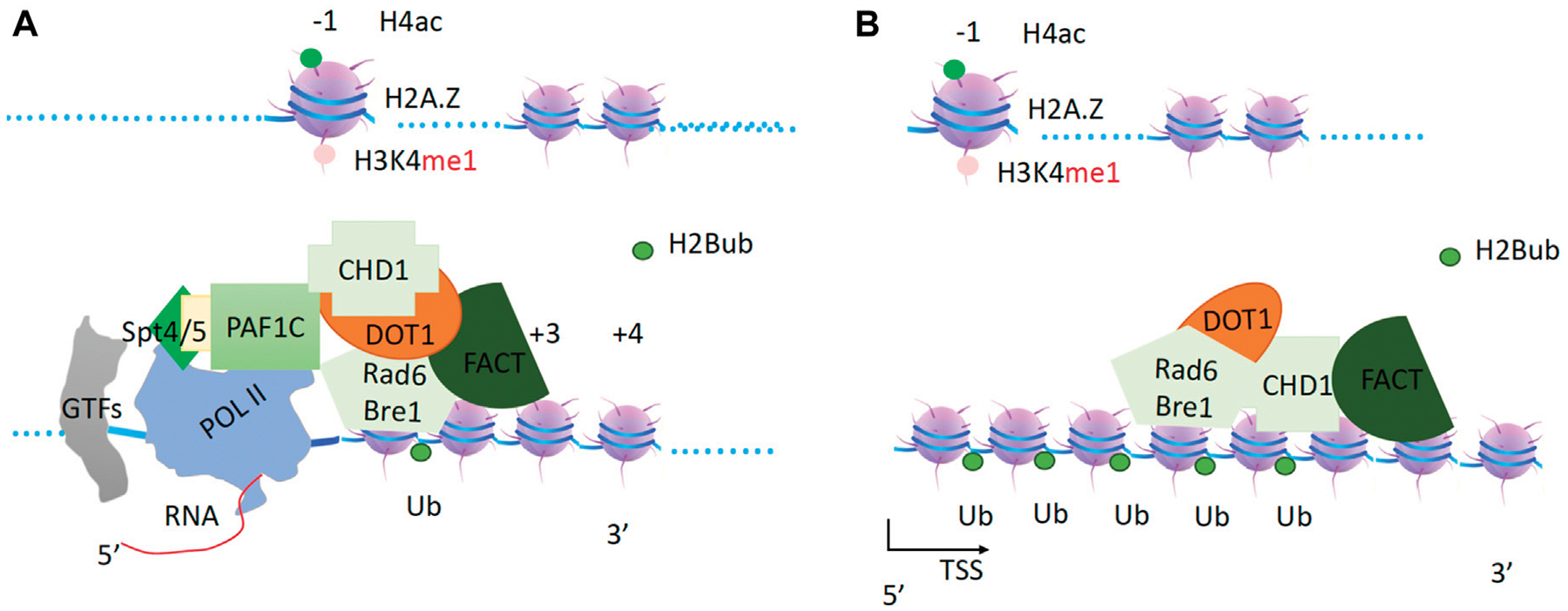
The generation of H2Bub and H3K79me3 within the gene bodies. **(A)**. Paf1c recruits CHD1, which in turn recruits FACT, Rad6/Bre1 complex, and Dot1. FACT as a processivity factor coupling with chaperone function. **(B)**. CHD1 drives the complex to carry out correspondent modifications to set up the boundary of a transcription unit.

Ubiquitination of H2B by Rad6 is essential for transcriptional activities (Robzyk et al., 2000), as further demonstrated in *GAL1* activation assays (Kao et al., 2004). H2B ubiquitination has been shown to be essential for Set1 function through its Swd2 subunit in yeast (Dover et al., 2002) and Wdr82 subunit in higher eukaryotes (Wu et al., 2008). H2B ubiquitination is found only within gene bodies (Xiao et al., 2005), and it was recently confirmed that H2B ubiquitination acts as a docking site for the Set1 complex (Xue et al., 2019) and Dot1 (Anderson et al., 2019), suggesting that it occurs prior to transcription elongation. Upon binding of transcription factors (TFs) upstream of the transcription start site and downstream of nucleosomes at the −1 position (Gill and Ptashne, 1987), TFs recruit the Mediator (Flanagan et al., 1991; Thompson et al., 1993), which in turn recruits Pol II to the promoter region with the help of general transcription factors (GTs) (Matsui et al., 1980; Buratowski et al., 1989). The Mediator also recruits the Srb10 (CDK8 in higher eukaryotes) module or Kin28 (CDK7 in higher eukaryotes) to phosphorylate the Ser5-CTD of Pol II (Hengartner et al., 1995; Kuchin et al., 1995; Liao et al., 1995), which generates binding sites for capping enzymes and the Bur1/2 complex (Qiu et al., 2009). Upon phosphorylation of the Ser5-CTD of Pol II (Ser5p-CTD Pol II) by Srb10/CDK8 or Kin28/CDK7, Mediator is released from Pol II. The Bur1/2 complex is recruited by Ser5p-CTD Pol II through its C-terminal interaction domain (CID) (Qiu, Hu, and Hinnebusch, 2009) and phosphorylates the linker region between the CTD and the body of Pol II (Rbp1) (Chun et al., 2019) as well as the C-terminal repeats of Spt5, which is recruited by newly synthesized RNA. Phosphorylated Spt5 recruits Paf1c (Qiu et al., 2006; Qiu et al., 2012; Mayekar et al., 2013; Wier et al., 2013). DSIF (Spt4/5 complex) is essential for the recruitment of PAF1 complex (PAF1c) (Qiu et al., 2006) (Figure 4).

The coordination of Pol II, DSIF, PAF1c, and RNA is further verified by the recent complex structure of Pol II-DSIF-PAF1c-Spt6 (Vos et al., 2018). PAF1c is an important platform for the recruitment of different cofactors. First, it recruits chromodomain helicase DNA binding protein 1 (CHD1), FACT (facilities chromatin transcription) complex (Simic et al., 2003; Pavri et al., 2006; Lee et al., 2017), and RAD6/Bre1 complex (Van Oss et al., 2016) (Figure 4). DOT1 (Disruptor of telomeric silencing 1) (Singer et al., 1998), which methylates H3K79 to prevent the heterochromatin formation and marks the zone of an active gene body (Feng et al., 2002; Lacoste et al., 2002; van Leeuwen et al., 2002), was found to associate with PAF1c and elongation complexes (Krogan et al., 2003a). DOT1 is thought to couple with CHD1, FACT, and the RAD6/Bre1 complex and to function immediately after RAD6, although data on the direct interactions between DOT1 and the CHD1 complex are not yet available. It has been reported that the chromodomains of CHD1 from yeast and higher eukaryotic CHD3/4 do not bind to any form of H3K4 tails, but the chromodomains of human CHD1 do bind to H3K4me3 (Sims et al., 2005). Another report showed that the chromodomains of CHD1 are not essential for CHD1 function in flies (Morettini et al., 2011). Nevertheless, a report showed that chromatin remodelers, including CHD1, INO80, SWI/SNF, RSC, and ISW1b, were found to associate with entire gene units, from promoters to gene bodies (Gkikopoulos et al., 2011; Yen et al., 2012; Narlikar et al., 2013). More specifically, chromodomain helicase DNA binding protein 1 (CHD1) (Delmas et al., 1993) was found to associate with entire active transcribing gene units along with gene bodies (Gkikopoulos et al., 2011), especially the FACT (facilities chromatin transcription) complex (Simic et al., 2003; Jeronimo et al., 2021). This is also true for the FACT complex (Pavri et al., 2006). One report showed that CHD1 is essential for Rad6/Bre1 function (Lee et al., 2012). Taken together from these published data, I propose that CHD1 acts as the sole engine or motor that drives FACT, Dot1, and Rad6/Bre1 to carry out their respective enzymatic activities on nucleosomes along gene bodies, starting from the nucleosome at +1 (Figure 4), with FACT acting as both a chaperone and a processivity factor to relax the tails of the histones and help Rad6/Bre1 to function.

## RSC carries NuA4 to acetylate H2A and H4 along gene bodies

After CHD1 (carrying DOT1 and RAD6/Bre1) leaves the transcription start site, what modification complex follows? Direct evidence came from a report showing that a transient burst of Esa1 (from NuA4, “nucleosomal acetyltransferase histone H4“) dependent H4K8 acetylation occurs immediately after *MET16* activation (Morillon et al., 2005). NuA4 acts primarily on H2A and H4 along gene bodies (Grant et al., 1997; Smith et al., 1998; Clarke et al., 1999; Ginsburg et al., 2009). Based on a recent report, the pan acetylation of H2A and H4 within gene bodies is generated by NuA4 (Martin et al., 2021). Questions remain as to how NuA4 is recruited, and how NuA4 exerts its function along gene bodies. There is a chromodomain of Esa1 (Tip60 in humans) that specifically recognizes the monomethyl group of H3K4me1 on the −1 nucleosome (Jeong et al., 2011). It was found that neither Set1 nor Set2 affects NuA4 recruitment (Ginsburg et al., 2009), suggesting that NuA4 could only be recruited by the monomethyl of H3K4, which is a mark on −1 nucleosomes of active promoters of genes prior to Set1 and Set2 recruitment. Recently, it was reported that transformation/transcription domain-associated protein (TRRAP) is essential to recruit NuA4 complex (Detilleux et al., 2022).

**FIGURE 5:**
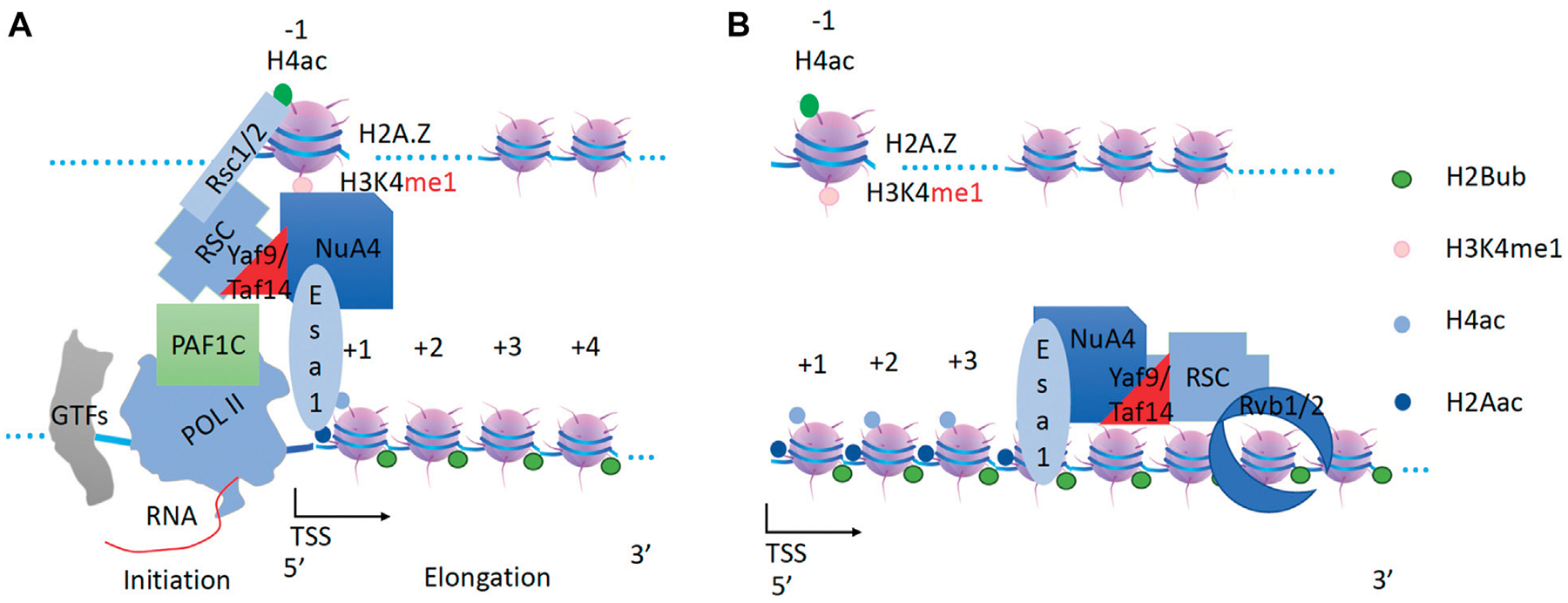
Generation of pan-acetylation of H2A and H4 on nucleosomes within the gene body by the RSC and NuA4 super-complex. **(A)** The chromodomain of Esa1 of the NuA4 subunit may help to recruit NuA4 to the promoter nucleosome through H3K4 with essential monomethylation (H4K4me1) at the −1 nucleosome. RSC associates with NuA4 through Yaf9 ot Taf14 subunit. **(B)** RSC will drive NuA4 along the gene body to acetylate H4 and H2A along the gene body.

On the other hand, the NuA4 complex requires an engine or a motor to exert its function along gene bodies. One form of NuA4 has been reported to form a super-complex with SWR1 in yeast (Keogh et al., 2006; Altaf et al., 2010; Wang et al., 2018). However, knockout of SWR1 does not result in a severe phenotype in yeast (Barbaric et al., 2007), while knockout of *Esa1*, the enzymatic component of NuA4, is lethal and it is an essential gene in yeast (Smith et al., 1998). This suggests that there must be some other remodeler(s) that supports a different form of NuA4. The YEATS-domain-containing protein Yaf9 was found to be required to bring NuA4 and SWR1 together (Wang et al., 2018). Interestingly, another YEATS domain-containing protein, Taf14, was found to associate with several complexes including three remodelers, RSC, SWI/SNF, and INO80 (Welch et al., 1993; Cairns et al., 1996a; Du et al., 1998; Eberharter et al., 1998; Sanders et al., 2002; Shen et al., 2003). I try to speculate that one of the three remodelers could act as an engine for NuA4 with Taf14 and Yaf9 as a notch (via YEATS-YEATS trimerization), since the YEATS domain of Yaf9 could form a trimer (Wang et al., 2009a; Li et al., 2014). A recent report showed that the ET (extra-terminal) domain within Taf14 could make specific interactions with *S*th1 (RSC), Snf5 (SWI/SNF), INO80, Sas3(NuA3), Taf2(TFIID), and Tfg1(TFIIF) (Chen et al., 2020). The RSC (Remodels Structure of Chromatin, PBAF in higher eukaryotes) complex, an ATP-dependent chromatin remodeling complex with homology to SWI/SNF (Cairns et al., 1996c; Cairns et al., 1999), could be recruited to the promoters by the −1 nucleosome through the bromodomain-rich subunits, *S*th1, Rsc2 or Rsc4 (Cairns et al., 1999). *In vitro* assays showed that both RSC and SWI/SNF could enhance the acetyltransferase activities of both NuA4 and SAGA (Carey et al., 2006), while RSC was found to localize along gene bodies (Ginsburg et al., 2009; Yen et al., 2012; Spain et al., 2014) as NuA4 (Grant et al., 1997; Smith et al., 1998; Clarke et al., 1999; Ginsburg et al., 2009), suggesting the potential for them to work together. It has also been reported that the BAH domain of RSC2 (a subunit of RSC, BAF180 in higher eukaryotes) binds to the H3K79 region in H3 (Chambers et al., 2013), suggesting that Dot1-methylated H3K79 may play a role in its recruitment along gene bodies. Most importantly, the knockout of either ESA1 of NuA4 (Smith et al., 1998) or *S*th1 of RSC (Cairns et al., 1999) is lethal in yeast. Interestingly, deletion of non-essential subunit(s) of RSC1/2 of RSC or mutation of ESA1 (non-lethal) has similar phenotypes as when the two mutations are combined (Ginsburg et al., 2009), suggesting that they function in the same pathway. Furthermore, combined mutations of Rsc1/2 deletion within RSC and gcn5/Spt3/Spt20 deletion within SAGA are synthetic lethal (Cairns et al., 1999), suggesting that RSC may not function in the same pathway as SAGA, although RSC enhances the activity of SAGA *in vitro* (Carey et al., 2006). From a temporal perspective, it has been shown that NuA4 activity at H4 is required for SWI/SNF recruitment, whereas gcn5 deletion has little effect on recruitment (Ginsburg et al., 2009), further suggesting that RSC acts before SAGA or SWI/SNF during transcriptional activation. Based on the above analysis, I attempt to speculate that RSC may drive NuA4 to acetylate H2A and H4 along gene bodies after their recruitment (Figure 5). TRA1 (TRRAP, transformation/transcription domain-associated protein, in higher eukaryotes), homologous to the ATM/PI-3 kinase but without kinase activity (McMahon et al., 1998), might act as a docking site to recruit both NuA4 and RSC. The Rvb1/2 complex (Jha and Dutta, 2009) could act as a mini-engine to rotate NuA4 and RSC around a specific nucleosome so that NuA4 can access all potential substrate sites on H4 histone tails.

## SWI/SNF couples with SAGA to acetylate H2B and H3 along gene bodies

It has been reported that H3K4me3 generation requires H2B ubiquitination (Dover et al., 2002; Ng et al., 2002; Sun and Allis, 2002) and occurs prior to H3 acetylation, which is coupled with H2B deubiquitination (Henry et al., 2003; Daniel et al., 2004). Prior to the process of transcription elongation, the nascent 5’ RNA generated by Pol II recruits Set1 (the H3K4 methyltransferase) through its RNA binding RRM domain (Luciano et al., 2017; Sayou et al., 2017), possibly with the help of PAF1c (Ng et al., 2003). The Set1 complex, with the help of the Spp1 subunit, converts H3K4me1 on the −1 nucleosome to H3K4me2/3 (Takahashi et al., 2009) after recruitment of NuA4 and RSC super-complex. The generation of H3K4me3 at promoter and enhancer regions is necessary for the further recruitment of transcriptional cofactors described below (Liu et al., 2020a).

The SAGA (Spt-Ada-Gcn-acetyltransferase) complex, which has been well-characterized as a histone acetyltransferase complex by several groups (Berger et al., 1992; Marcus et al., 1994; Brownell and Allis, 1995; Brownell et al., 1996; Grant et al., 1997), works specifically on H2B and H3. There is the Sgf29 subunit within SAGA that is specific for H3K4me3 on the −1 nucleosome (Schram et al., 2013). Based on the role of SAGA in the deubiquitination of H2B in the gene bodies (Henry et al., 2003; Daniel et al., 2004), it can be inferred that SAGA works along the gene body after NuA4. Indeed, SAGA has been found to act on coding regions to stimulate Pol II elongation (Govind et al., 2007; Wyce et al., 2007). Interestingly, SAGA was found to be required for the transcription of all expressed genes (Bonnet et al., 2014), and this conclusion was further verified a few years later (Baptista et al., 2017). However, this concept has been challenged by a recent report (Donczew et al., 2020). There is a need of further verification of the role of SAGA in transcription regulation. Again, there is no engine or motor within the SAGA complex to carry out the enzymatic function of SAGA along the gene bodies.

**FIGURE 6:**
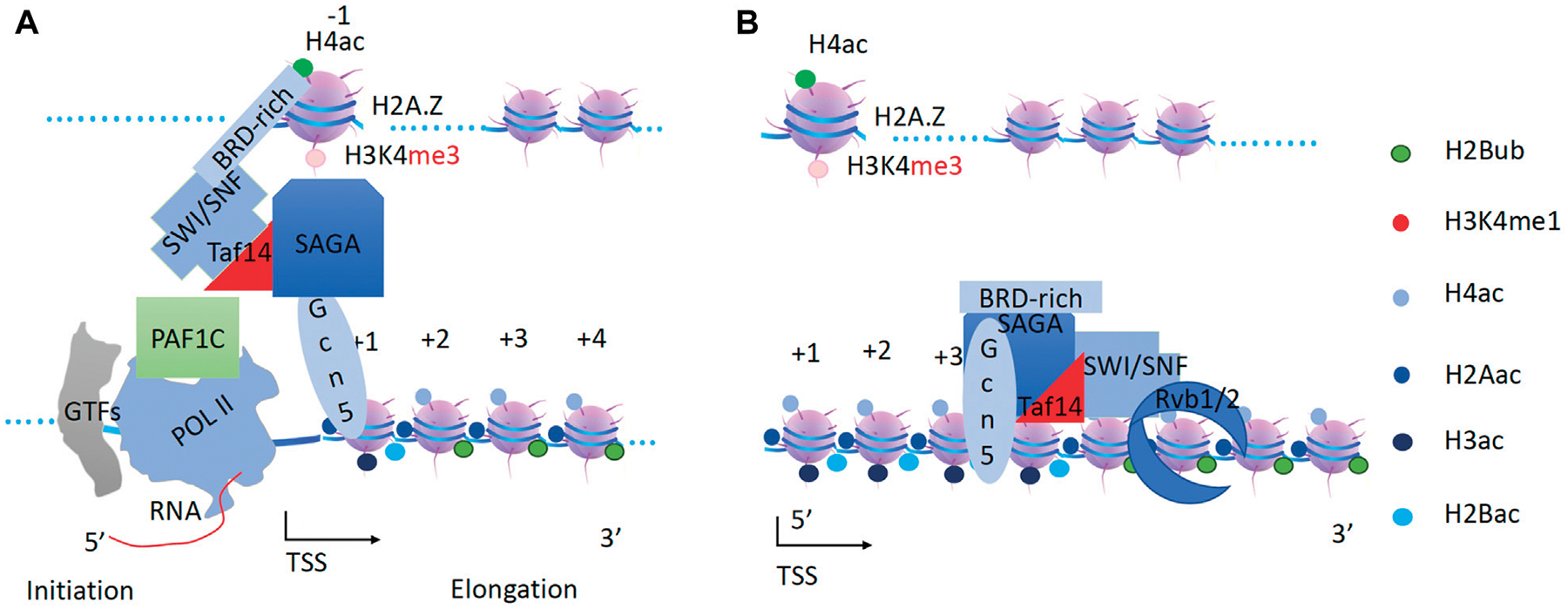
The generation of pan-acetylation of H2B and H3 of nucleosomes within the gene body by the SWI/SNF and SAGA super-complex. **(A)**. After H2A and H4 pan-acetylation, Set1 is recruited by Pol II to convert H4K4me1 to H3K4me3. Transcription factors recruit SWI/SNF to the promoter. SAGA complex is recruited to the −1 nucleosome through Sgf29 by H3K4me3 on the −1 nucleosome. SWI/SNF will associate with SAGA via Taf14. **(B)**. SWI/SNF will drive the SAGA complex along the gene body to acetylate all H3 and H2B coupled with de-ubiquitination of H2B.

On the other hand, the SWI/SNF complex (BAF complex in higher eukaryotes) was first characterized as a chromatin remodeler and regulates the transcriptional activities of a group of genes (Neigeborn and Carlson, 1984; Stern et al., 1984; Breeden and Nasmyth, 1987; Laurent et al., 1990; Peterson and Herskowitz, 1992). The SWI/SNF complex has been reported to be recruited by transcription factors (Yoshinaga et al., 1992; Cairns, Levinson, et al., 1996b; Fryer and Archer, 1998; Cosma et al., 1999; Kim et al., 1999; Krebs et al., 1999). An early study of the expression of the *HO* gene, an endonuclease expressed during the cell cycle, found that the transcription factor Swi5 first recruits SWI/SNF, which in turn recruits SAGA to the promoter region of *HO*; both complexes remain at the promoter for a period of 30–35 min (Cosma et al., 1999). A subsequent transcriptional activation analysis of the *PHO8* gene, which encodes a vacuolar alkaline phosphatase, showed that both SWI/SNF and SAGA are required for the hyperacetylation of H3 and H4 at promoter-proximal regions (Reinke et al., 2001). Interestingly, knockout of *snf2* of the ATPase subunit results in the intermediate accumulation of both acetylated H3 and H4, while either deletion or enzymatically inactive single mutant of *GCN5* abolishes the effects of hyperacetylation (Reinke et al., 2001), suggesting that loss of SWI/SNF function could lead to the stalling of SAGA at the promoter region of *PHO8*. Similar results were obtained from the experiments of transcriptional activation experiments of *PHO5*, a gene encoding an acid phosphatase, where both SWI/SNF and SAGA are recruited to the promoter and trigger the expression of *PHO5* (Dhasarathy and Kladde, 2005). Taken together, all these data suggest that SWI/SNF may couple with SAGA to deposit acetyl-groups on H2B and H3 along the gene bodies and simultaneously remove ubiquitin on H2B (Figure 6). Similar to RSC and NuA4 described in the previous section, TRA1 as docking site to recruit SAGA and SWI/SNF while Rvb1/2 could couple with the super-complex along the gene bodies. Furthermore, TAF14, the YEATS domain-containing protein, is found to associate with SWI/SNF (Cairns et al., 1996a; Sanders et al., 2002) while a homologous YEATS2 protein is found to associate with SAGA or ATAC complex in animals (Fasci et al., 2018; Kim et al., 2019), suggesting that TAF14 could be a connector between SWI/SNF and SAGA in yeast.

## The first round of Pol II transcription and H3K36me3 establishment by Pol II and Set2

After the acetylation of histone tails of nucleosomes inside gene bodies by NuA4 coupling with RSC and SAGA coupling with SWI/SNF, all nucleosomes along gene bodies are fully acetylated and ready for Pol II to elongate along gene bodies with fragile nucleosomes. The critical roles of RSC and SWI/SNF for the pioneering round of transcriptional activation could be indirectly demonstrated by the severe synthetic phenotype caused by the double mutant with a temperature-sensitive mutant of *S*th1 (the ATPase subunit of RSC) and the deletion of Snf2 for the expression of *PHO5* (Musladin et al., 2014), as well as the eviction of nucleosomes at the promoters of highly expressed genes through cooperative actions of RSC and SWI/SNF (Rawal et al., 2018).

**FIGURE 7:**
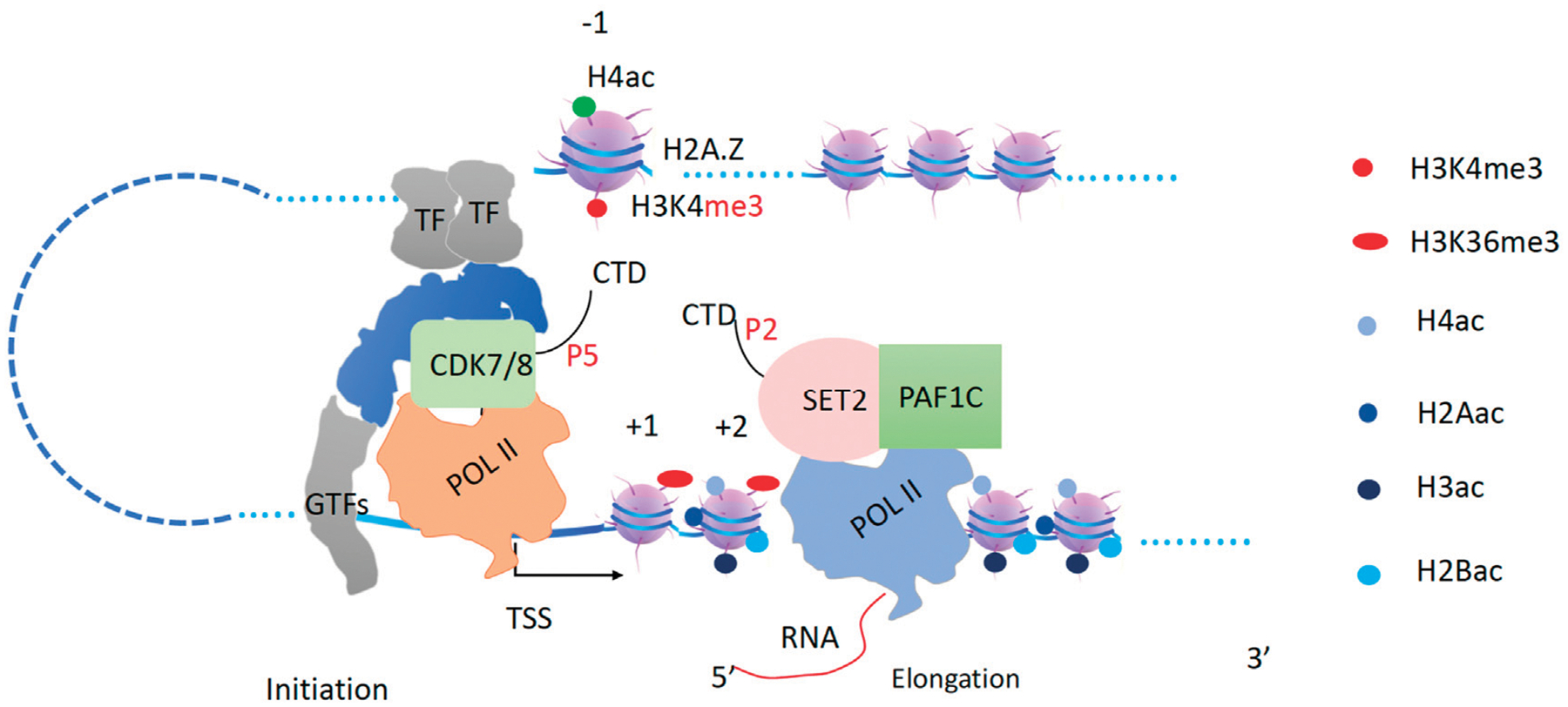
Transcription elongation and H3K36me3 generation by Set2. The pioneer Pol II begins to transcribe coupled with Set2 to generate H3K36me3 behind. TFs and Mediator with CDK7/8 complex will recruit the second Pol II.

The newly synthesized 5′RNA and the Ser5p-CTD (phosphorylated by the CDK8 module of Mediator) of Pol II will recruit the CTK1 complex (Muhlbacher et al., 2015; Battaglia et al., 2017) through a CTD binding domain of CTK3, which binds to Ser5p-CTD (Muhlbacher et al., 2015). The CTK1 (CDK12/13 in higher eukaryotes) complex phosphorylates the Ser2-CTD of Pol II to generate active Ser2p-CTD of Pol II (Lee and Greenleaf, 1991; Sterner et al., 1995; Hautbergue and Goguel, 2001; Bartkowiak et al., 2010) (Figure 7). Based on our recent discoveries, the Ser2p-CTD of Pol II generated by CTK1 in yeast and CDK12/13 in higher eukaryotes should be different from the Ser2p-CTD of Pol II generated by CDK9 (Liu et al., 2020b). The phosphorylated CTD (Ser2p-with or without Ser5p-CTD) of Pol II is required for the recruitment of Set2, an H3K36 methyltransferase that methylates H3K36 along the coding regions with the help of Paf1c (Li et al., 2003). I propose that Set2 is bound to Pol II and methylates H3K36 on newly assembled nucleosomes after Pol II proceeds with transcription elongation (Figure 7). The Ser2p-CTD of Pol II is also required to recruit the polyadenylation complex, and the termination complex, etc. At the same time, transcription factors (TFs) and Mediator will recruit a second Pol II to the promoter region (Figure 7). After the first Pol II leaves, mixed nucleosomes with fully acetylated histones as well as naïve ones, all containing the H3K36 methylation, are rebuilt along gene bodies (Figure 7).

## SWR1 drives a sub NuA4 to acetylate newly engaged H2A and H4

After the pioneering round of transcription, there could be two forms of transcription elongation mechanisms, one is TFIID dependent for ~85–90% of genes in the yeast (Donczew et al., 2020; Rossi et al., 2021), which is beyond the scope of this essay. For the remaining genes (~10–15%) (Donczew et al., 2020; Rossi et al., 2021), I propose that they are SWR1 and INO80 dependent. Furthermore, I propose that the super-complex SWR1/NuA4(EAF3) is responsible for the pan-acetylation of H2A and H4 while INO80-NuA3 is responsible for H2B and H3 in the newly assembled nucleosomes after the first round of transcription.

**FIGURE 8:**
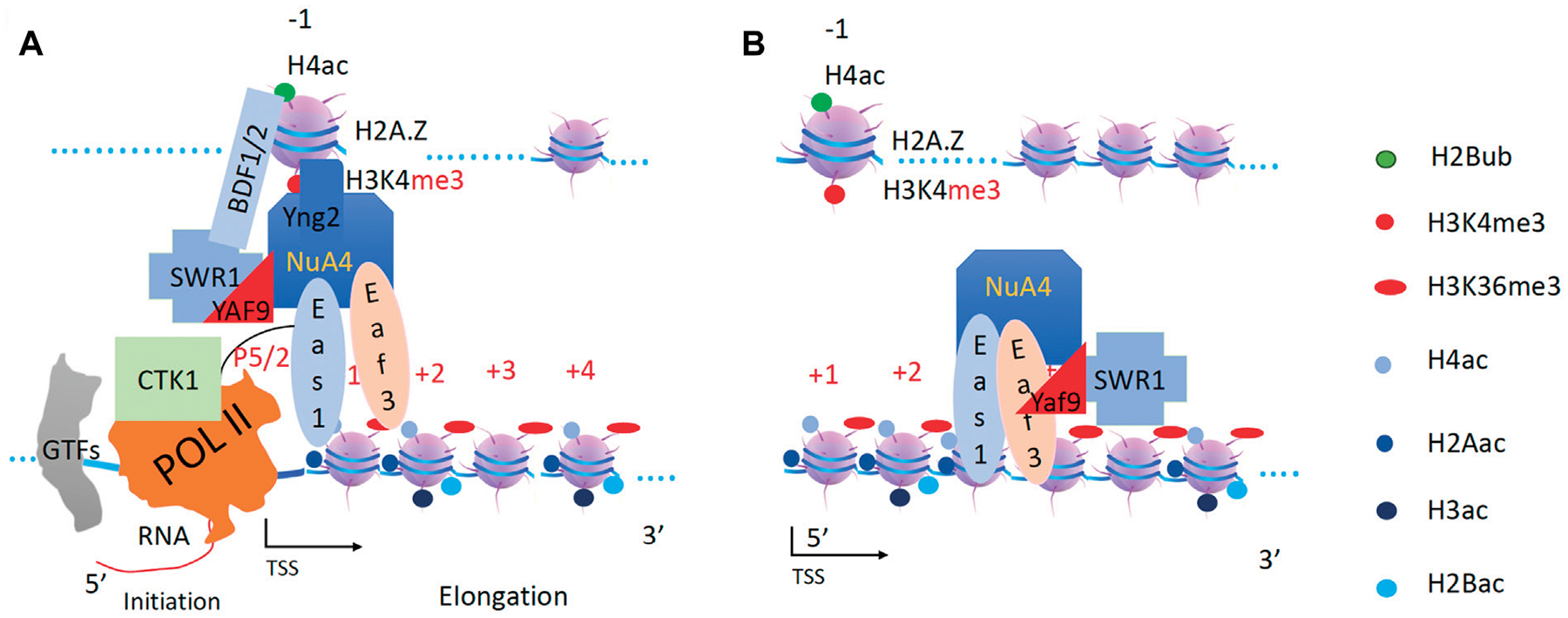
SWR1 and NuA4 are recruited and acetylate H2A and H4 on the newly assembled +1 nucleosome. **(A)**. NuA4 and SWR1 are recruited to the promoter region. **(B)**. SWR1 drives the second form of the NuA4 complex to function along the gene body.

The chromatin remodeling complex SWR1, originally defined as an exchanger of H2A.Z and H2A (Krogan et al., 2003b; Kobor et al., 2004; Mizuguchi et al., 2004), could be recruited to the −1 nucleosome containing pan-H4ac through its multiple bromodomain-containing subunit BDF1/2 (Ladurner et al., 2003). NuA4, which shares several subunits with SWR1 (Krogan et al., 2003b; Kobor et al., 2004; Mizuguchi et al., 2004) and has been shown to cooperate with SWR1 (Keogh et al., 2006). Interestingly, it was reported that SWR1 and NuA4 could interact through a bridging subunit YAF9 (GAS41 in higher eukaryotes), a YEATS domain-containing protein (Wang et al., 2018). It was further confirmed that SWR1 and NuA4 cooperate and associate with H2A.Z (Altaf et al., 2010), suggesting that the super-complex is first recruited by the −1 nucleosome since H2A.Z prefers to reside in nucleosomes at the promoter region (Figure 8A). Furthermore, SWR1 and its related complex INO80 were found to be distributed not only at promoter regions but also along the gene body (Yen et al., 2012), suggesting that the association of H2A.Z with SWR1 is only the beginning of a longer process. There are two forms of NuA4 complexes that exist *in vivo*: NuA4 and Piccolo NuA4 (Boudreault et al., 2003), the latter containing Yng2, the H3K4me3 binding protein; and the Eaf3 subunit (MRG15 in higher eukaryotes), which contains a PWWP domain that recognizes H3K36me3. Interestingly, the PWWP-domain-containing subunit of Eaf3 of the variant NuA4, specific for H3K36me3, has been reported to associate with SWR1 in yeast, whereas MRG15 of the Tip60 complex associates with p400 or SRCAP (homologs of SWR1 in mouse or human) (Doyon et al., 2004; Auger et al., 2008). This unique feature of PWWP domain subunit-coupled complexes of either in Yeast (SWR1) or higher eukaryotes (p400 or SRCAP) suggests that the super-complex of SWR1 and variant NuA4 works on later rounds of transcription after deposition of H3K36me3 by the first round of Set2 and Pol II. I believe that the variant form of NuA4 is recruited by H3K4me3 on the −1 nucleosome and H3K36me3 on nucleosomes within the gene bodies. This unique form of NuA4 is driven by SWR1 to perform pan-acetylation of all H2A and H4 of newly integrated nucleosomes along the gene body (Figure 8B). Interestingly, deletion of SWR1 barely perturbs the transcriptional activity of yeast genome (Barbaric et al., 2007), suggesting that the super-complex of RSC and NuA4 could cover the function of the super-complex of SWR1 and NuA4 when needed (redundant) or the mixed nucleosome composition could still be overcome by Pol II although it might be not as efficient.

Due to the uncertainty of where the newly formed naïve nucleosomes are located, pan-acetylation could occur at the +1 nucleosome or any downstream nucleosome, such as the +4 nucleosome shown here (not shown). Due to these random placements of naïve nucleosomes, which (using these super-complexes) will move the −1 nucleosome (to recruit the corresponding super-complex) downstream of the TSS, the downstream distribution of H3K4me3 along gene bodies reported by the H3K4me3-ChIP-seq data or the two peak distribution flanking the transcription initiation site occurs.

## INO80 brings NuA3 to acetylate H2B and H3

Similar to the pioneering round of histone acetylation modification, after pan-acetylation of H2A and H4 by the super-complex of SWR1 and NuA4, there is a need for the pan-acetylation of H2B and H3 within the newly assembled nucleosomes. From the remodeler aspect, INO80 (inositol-requiring mutant 80), a remodeler, was first identified in the yeast (Ebbert et al., 1999; Shen et al., 2000) and conserved in the mammals (Jin et al., 2005), associated with nucleosomes of the entire coding region (Yen et al., 2012). The bromodomain-rich protein Bdf1 has been reported to associate with INO80 at the promoter region (Yen et al., 2013), suggesting the potential recruitment of INO80 to the −1 nucleosome with pan-acetylation of H4. Deletion of INO80 slightly affects the transcriptional activity in the yeast (Barbaric et al., 2007), more specifically, ~10% of genes across the genome are affected in the yeast (Qiu et al., 2020). Considering the function of all remodelers assigned in the previous sections, there must be an H2B- and H3-specific acetyltransferase coupling with INO80.

**FIGURE 9:**
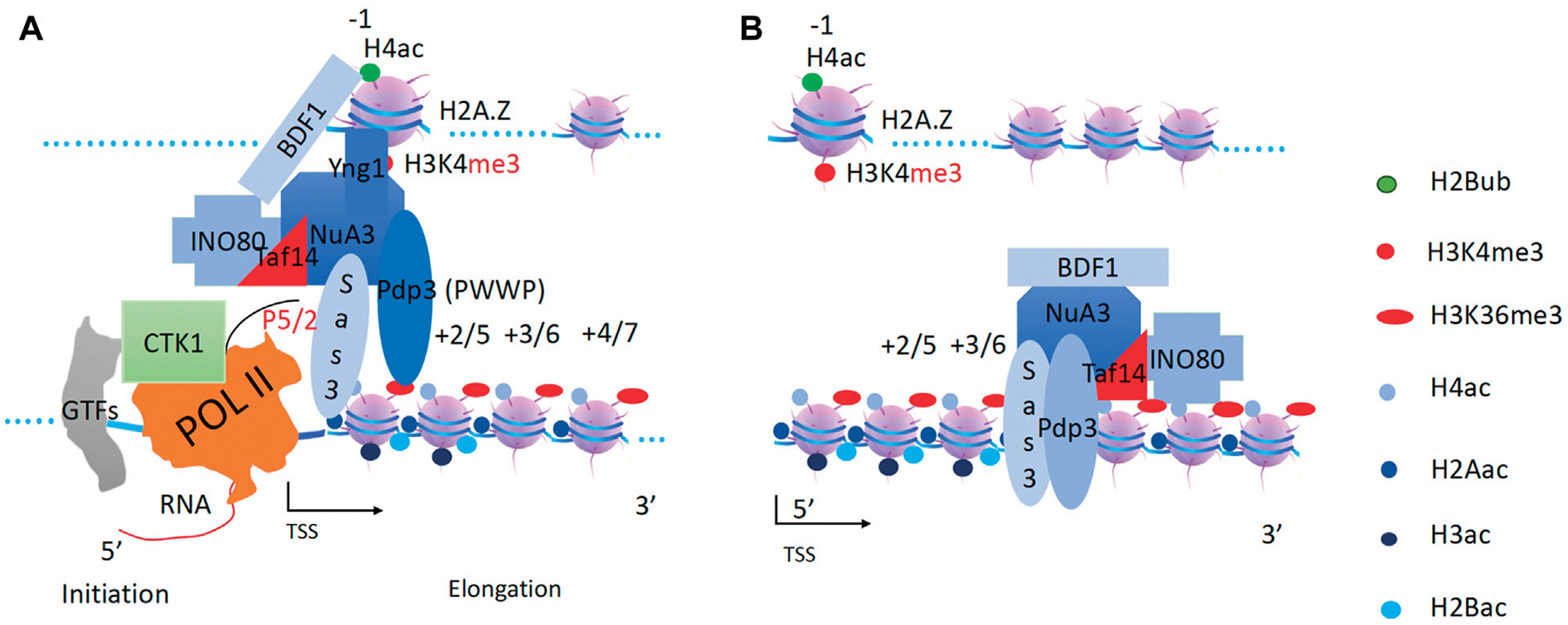
INO80 couples with the NuA3 complex to acetylate H2B and H3 on newly assembled nucleosomes. **(A)**. In higher eukaryotes, the MYST2 complex (BRPF1, ING5, EAF6) acts as NuA3 to function on H3 and possibly H2B. BRPF1 contains PHD domain, bromodomain, and PWWP domain. Nto1 + pdp3 (subunits in yeast NuA3 complex) = BRPF1 (in human). **(B)**. INO80 drives NuA3 to carry out the acetylation on H2B and H3.

Conversely, NuA3 (nucleosomal acetyltransferase of histone H3), which has been characterized as an H3-specific acetyltransferase complex (John et al., 2000), may join INO80 and function through the Yng1 subunit (which binds to H3K4me3), given that Taf14 allows both INO80 and NuA3 to interact with each other. NuA3 acts similarly to SAGA (but without the H2B deubiquitinase module) to acetylate all H2B and H3 histones with the additional help of H3K36me3, which attracts the NuA3 complex through the PWWP domain-containing Pdp3 subunit (BRPF1 in higher eukaryotes) (Gilbert et al., 2014) (Figure 9). A recent report confirms that the activity of NuA3 is coupled to Set2 (Georgescu et al., 2020). Interestingly, it has been reported that a synthetic lethal phenotype was reached only with deletion of both Gcn5 and Sas3 (the enzymatic subunit of NuA3) (Howe et al., 2001), suggesting that acetylation of H2B and H3 is essential for transcription and that Gcn5 and Sas3 play complementary roles.

However, direct interaction data between INO80 and NuA3 has been lacking until recently. In higher eukaryotes, there are several groups of MYST complexes, named from human MOZ (monocytic leukemia zinc finger protein) (Borrow et al., 1996), yeast Ybf2/Sas3 (something about silencing 3), and Sas2 (Reifsnyder et al., 1996), mammalian TIP60 (HIV Tat-interacting protein 60 kDa) (Kamine et al., 1996). Myst2 was found to be pulled down with INO80 by the transcription factor Niam (Pardo et al., 2017). Proteomic analysis of the pulldown by Hap2, an HMG box protein in *S. Pombe* and a component of INO80, found that NuA3 coexists with INO80 (Singh et al., 2020). More interestingly, INO80 was found to be highly associated with H3 pan-acetylation, whereas deletion of INO80 leads to a dramatic decrease of acetylated H3K18 (Beckwith et al., 2018). These recent findings suggest that INO80 works in concert with NuA3 to acetylate H3 and possibly H2B along gene bodies (Figure 9). After pan-acetylation of newly assembled nucleosomes by both NuA4 and NuA3, the second Pol II is ready for transcription elongation, just like the first Pol II and all subsequent Pol IIs.

Compared to the lethal phenotype caused by the single deletion of Eas1, the acetyltransferase subunit in NuA4 (Smith et al., 1998; Allard et al., 1999; Clarke et al., 1999), double deletion of Gcn5 (the acetyltransferase subunit of SAGA) and Sas3 (the acetyltransferase subunit of NuA3) also causes lethality in yeast (Howe et al., 2001) as mentioned above. Gcn5 and Sas3 are responsible for the pan-acetylation of H2B and H3, whereas Esa1 alone is responsible for the pan-acetylation of H2A and H4. These lethal phenotypes, caused by the deletion of either the pan-acetylated complexes of H2B and H3, or H2A and H4, suggest that both mechanisms are required and essential for pol II to transcribe along coding regions.

## ISW1b buddies with RPD3s to remove acetyl-groups on H2A and H4

When inhibitory signals arrive or transcription factors dissociate, the transcription unit begins to shut down and all nucleosomes should be reset for the next stimulation. Based on the above analysis of transcription elongation: all histone modification complexes are driven by a remodeling engine or Pol II, I believe the same is true for histone deacetylases working on coding regions along the gene body. Several reports show that the deletion of both CHD1 and ISW1b leads to the failure of nucleosome rebuilding and cryptic transcription initiation sites within gene bodies in yeast (Quan and Hartzog, 2010; Gkikopoulos et al., 2011; Smolle et al., 2012). I propose that CHD1 and ISW1b are required for the recruitment of histone deacetylase complexes to remove all acetyl groups along the gene bodies. Without the deacetylation process supported by CHD1 and ISW1b, pan-acetylated nucleosomes are fragile and easily fall apart (ejected), exposing naked DNA for Pol II binding as well cryptic transcription initiations.

**FIGURE 10:**
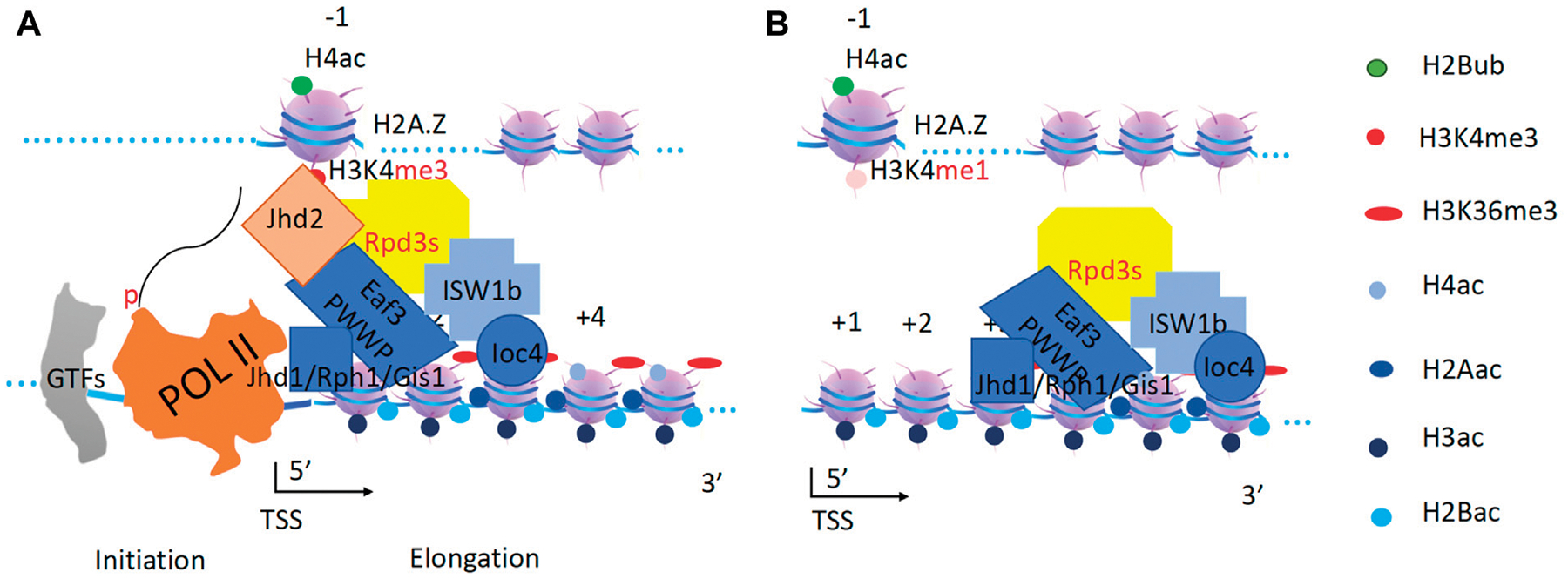
Resetting of H2A and H4 by ISW1b and Rpd3s complex. **(A)**. Rpd3s removes all acetyl-groups from H2A and H4. Eaf3 is a mammalian homolog of MRG15. Jhd2 converts H3K4me3 to H3K4me1 at the promoter (−1 nucleosome). Jhd1/Rph1/Gis1 convert H3K36me3 to H3K36me0 along the gene body. **(B)**. ISW1b drives both deacetylase and demethylases along gene bodies to carry out their correspondent functions.

ISW1b is known to be distributed throughout the genome (Yen et al., 2012). ISW1b contains an Ioc2 subunit, which can recognize H3K4me3 on −1 nucleosome, and an Ioc4 subunit with PWWP specific for H3K36me3 recognition (Smolle et al., 2012), which may help ISW1b bind to nucleosomes along the gene body. The ISW1b homolog, ISWI, in higher eukaryotes has been found to associate with Sin3 and Rpd3 complex containing HDAC1/2 (Burgio et al., 2008). Sin3 and Rpd3 are normally coupled to each other (Zhang et al., 1997b; Zhang et al., 1998). Rpd3s is characterized as a histone deacetylase that acts on acetylated H4 (De Rubertis et al., 1996; Vannier et al., 1996; Sun and Hampsey, 1999; Kurdistani et al., 2002). Furthermore, Rpd3s function is always coupled to Set2 through the H3K36me3-binding subunit Eaf3 (Carrozza et al., 2005; Keogh et al., 2005), where Set2 adds methyl groups to H3K36 within the gene body to create a docking site for Eaf3. Based on this information, it appears that ISW1b is recruited and coupled to the Rpd3s complex to perform deacetylation within coding regions. It is assisted by both the H3K36me3 binding subunit Eaf3 (PWWP domain) of the Rpd3s complex (Carrozza et al., 2005; Keogh et al., 2005) and Ioc4 from ISW1b (Smolle et al., 2012), which removes all acetyl groups on H2A and H4 histones (Robyr et al., 2002) (Figure 10).

Interestingly, a highly conserved ER (Esa1-Rpd3) motif was found within the catalytic domains of both ESA1 and Rpd3 (Adachi et al., 2002), suggesting that they share substrates though opposite effects (writer and eraser). In addition, it was found that RSC and ISW1 were found to counteract each other as antagonists to regulate promoter opening (Parnell et al., 2015), another direct strong piece of evidence supporting the coupling of RSC and NuA4, as I proposed earlier.

In higher eukaryotes, RBP2, an H3K4me3 demethylase, is coupled to the Sin3-Rpd3 complex (Hayakawa et al., 2007). In yeast, the ISW1b-Rpd3 super-complex is coupled to Jhd2 (Seward et al., 2007) to convert H3K4me3 back to H3K4me1 (Figure 10). In higher eukaryotes, the HDAC3 complex NCoR/SMRT is coupled with JMJD2, an H3K36me3 demethylase (Yoon et al., 2003). There are similar demethylases (Jhd1, Rph1, and Gis1) that may be coupled to ISW1 in yeast (Chen et al., 2006; Tsukada et al., 2006; Whetstine et al., 2006; Tu et al., 2007). ISW1 has been reported to interact with Jhd1 (Babour et al., 2016), and Jhd1 and Rph1 have been reported to associate with each other, suggesting the complete removal of methyl groups on H3K36me3 (Sein et al., 2015). ISW1b is recruited and couples with Rpd3s, Jhd2, and Rph1/Jhd1/Gis1 complexes. After H3K4me3 is converted to H3K4me1 on −1 nucleosomes, Jhd2 can fall off. ISW1b will drive Rpd3s along gene bodies to remove all acetyl groups on H2A and H4, while the Rph1/Jhd1/Gis1 complex will remove methyl groups on H3K36 (Figure 10). Rpd3L can also be recruited and coupled with ISW1a or ISW2 to remove acetyl groups on the H4 histone on −1 nucleosomes, also for complete resetting (Lee et al., 2018). In higher eukaryotes, H4 acetylation has been shown to directly counteract chromatin compaction mediated by ISWI (homolog of ISW1a), as evidenced by the coupled function between remodelers and histone deacetylases, suggesting specific resetting at the promoter region (Corona et al., 2002).

## CHD1 assists HDA1 to deacetylate H2B and H3

**FIGURE 11:**
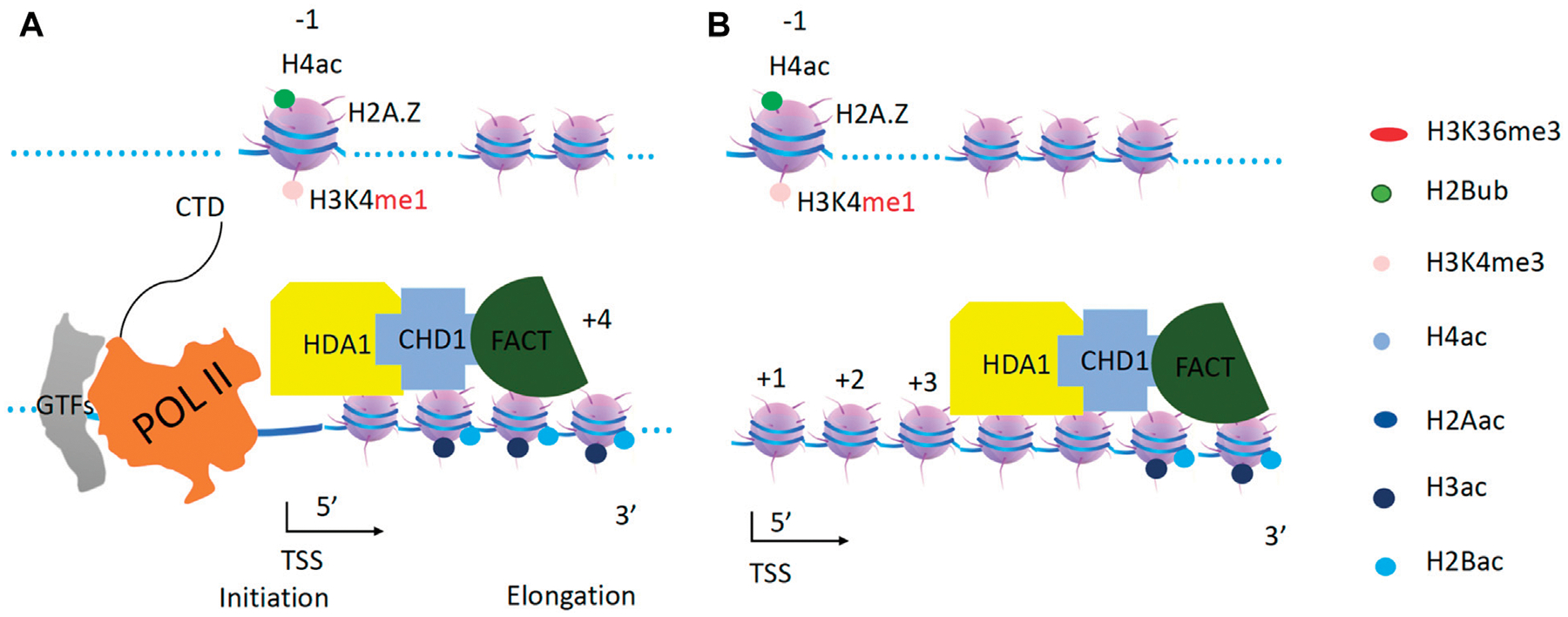
CHD1 and HAD1 function on acetylated H2B and H3. **(A)**. Both HDA1 and CHD1 are recruited to the promoter. **(B)**. CHD1 with the help of FACT drives HDA1 along gene bodies to carry out deacetylation to reset the transcription unit.

As mentioned above, both CHD1 and ISW1b contribute to cryptic transcription and nucleosome rebuilding (Quan and Hartzog, 2010; Gkikopoulos et al., 2011; Smolle et al., 2012). CHD1 is required to drive Rad6/Bre1 and Dot1 along the gene body before the first round of transcription, as it was discussed earlier. It is unlikely that H2B ubiquitination (loss of H2B ubiquitination due to CHD1 deletion), which is eliminated after the first round of transcription, or H3K79 methylation (no methylation due to CHD1 deletion), which prevents heterochromatin formation, will contribute to cryptic transcription and nucleosome rebuilding. In higher eukaryotes, there exists a NuRD complex, containing the remodeler CHD3/4, that acts as a histone deacetylase to shut down the transcription (Tong et al., 1998; Xue et al., 1998; Zhang et al., 1998a). Interestingly, neither the chromodomains from CHD1 of yeast nor those of higher eukaryotic CHD3/4, bind to any form of H3K4 (Sims et al., 2005). I propose that CHD1 from *Saccharomyces cerevisiae* (with homologs of CHD3/4) plays a similar role as CHD3/4 in the NuRD complex to bring a histone deacetylase complex to shut down transcription, in addition to its role in assisting H2B ubiquitination and H3K79 methylation at the first round of transcription. Interestingly, a single knockout of CHD1 results in a dramatic accumulation of acetylated H3 (Quan and Hartzog, 2010), suggesting that CHD1 may couple with histone deacetylase(s) other than Rpd3s, which primarily targets H4 (Robyr et al., 2002). In the same report, HDA1, a class II histone deacetylase found in yeast, is responsible for deacetylating H3 and H2B (Robyr et al., 2002). A later report showed that HDA1 targeting of H2B and H3 has an antagonistic effect on Gcn5 (Islam et al., 2011). Data from S. *Pombe* showed that a NuRD ortholog, SHREC, contains Mit1 and Clr3 (ortholog of HDA1 in budding yeast) to silence active transcription and support heterochromatin formation (Job et al., 2016). One puzzle that needs to be resolved is that NuRD in higher eukaryotes containing HDAC1/2, a class I deacetylase similar to Rpd3 instead of HDA1, a class II deacetylase. Although no HDA1 ChIP-seq data are available, one report shows that HDAC6 (and HDAC4,5,7, etc., which belong to the class II histone deacetylase family in higher eukaryotes, such as HDA1 in yeast) is mostly found along the gene body (Wang et al., 2009b). It is likely that CHD1 and HDA1 are recruited together. CHD1 with the help FACT (acting as a processivity factor) drives HDA1 along the gene body to deacetylate H3 and H2B (Robyr et al., 2002). HDA1 and Rpd3s work complementarily with one another to remove all acetyl groups (Rundlett et al., 1996). After these two rounds of deacetylation, the nucleosomes on the gene body are reset (Figure 11).

## Experiments needed to verify the hypothesis

**FIGURE 12:**
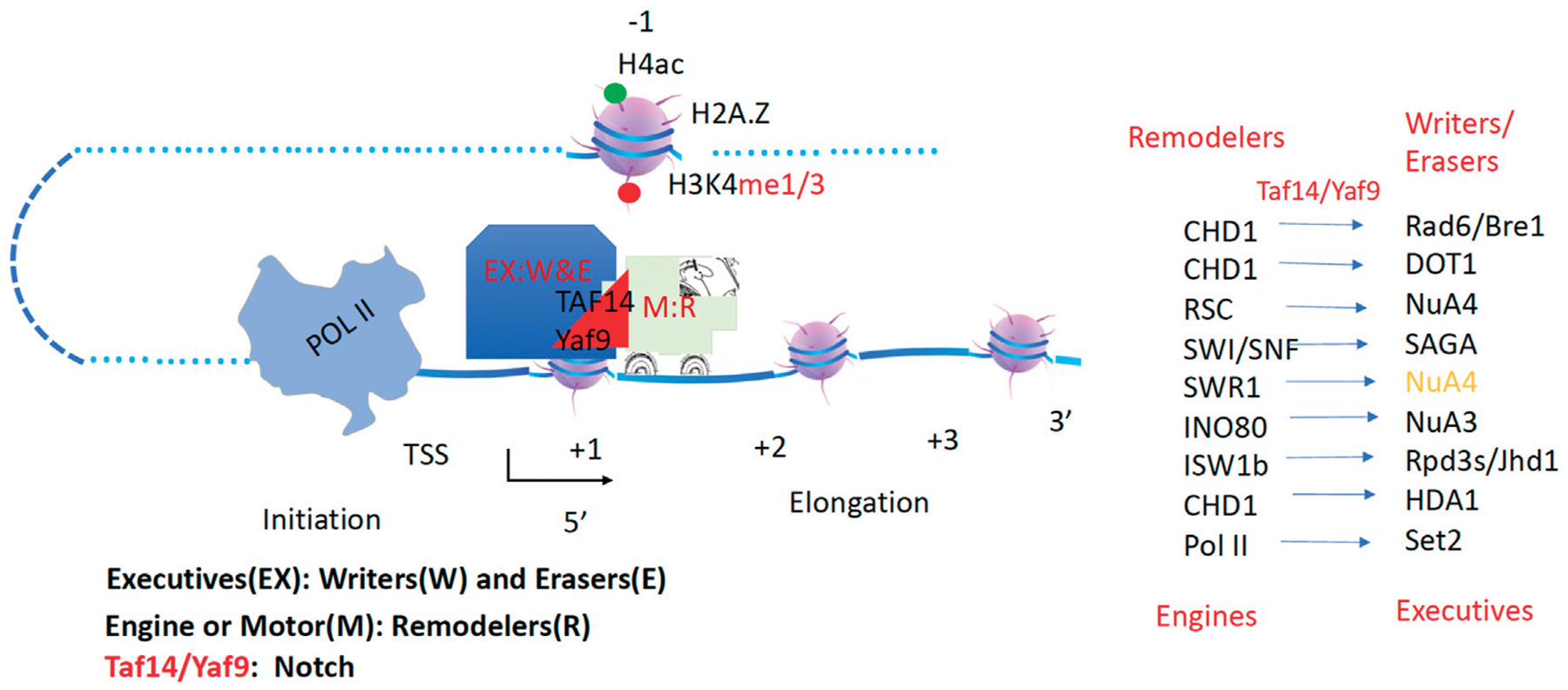
The overall model of transcription. Engines or motors (Remodelers) carry cargos or executives (Writer/Erasers) to function along the gene body before or after Pol II transcription. Yaf9/Taf14 acts as a notch.

In this essay, due to length limitation, I only focus on a list of remodelers and their potential cargos to integrate their function into the overall transcriptional activation and elongation involved in Pol II (Figure 12). As shown in an elegant report, SAGA is required for the transcription of almost all genes, as mRNAs are decreased after deletion of *spt3/spt7/spt20*, whereas only ~13% genes are affected when *spt3/spt7/spt20* was rapidly depleted by a Degron system instead of deletion from knockout in yeast (Donczew et al., 2020). On the other hand, acute depletion (knockout is lethal) of individual TFIID components, Taf1/Taf7/Taf13, causes dramatic reductions in ~87% of genes, but mild reductions to the ~13% of genes (Donczew et al., 2020). The results are slightly different from another report (Rossi et al., 2021) in which a group of TFIID-dependent genes are constitutively active and completely independent of SAGA. Another interesting report with a series of complex structures of TFIID and TATA-containing and TATA-less promoters showed that TFIID seems to bind to both promoters without discrimination (Chen et al., 2021). To reconcile these excellent data, it can be explained by the context of this essay. In the pioneer round (a first mechanism), most genes are controlled by RSC-NuA4 and SWI/SNF-SAGA. For the second and subsequent rounds of transcription, two regulatory mechanisms are followed. ~13% of stimulation-dependent genes are controlled by SWR1-NuA4 and INO80-NuA3 (a second mechanism), while ~87% of housekeeping genes (including constitutively active genes) are regulated by TFIID in the second and subsequent rounds (a third mechanism). The detailed regulatory mechanism of TFIID is beyond the scope of this essay and will be analyzed elsewhere, while there is a report suggesting that TFIID is involved in the transcription of second and subsequent rounds (Joo et al., 2017). On the other hand, all these recent data strongly support the novel concept presented in this essay. However, there are still several pair assignments that require further biochemical and genetic data to be finally confirmed. First, although abundant data have been found to support that RSC and NuA4 function together, direct interaction or association of the two complexes is still lacking. It may be due to the weak and fast dynamics of their cooperative actions for people to catch, or due to the lack of interest and artificially omitted for further characterizations. This is also true for the super-complex of SWI/SNF and SAGA, as well as the super-complex of INO80 and NuA3. Second, why are there three transcription regulation mechanisms (I: RSC, SWI/SNF, NuA4, and SAGA dependent; II: SWR1, INO80, NuA4, and NuA3 dependent; III: TFIID dependent), and what are the differences and similarities of initiation among these three mechanisms? Third, what is the exact mechanism of TFIID-dependent transcriptional regulation?

## Conclusion

**FIGURE 13:**
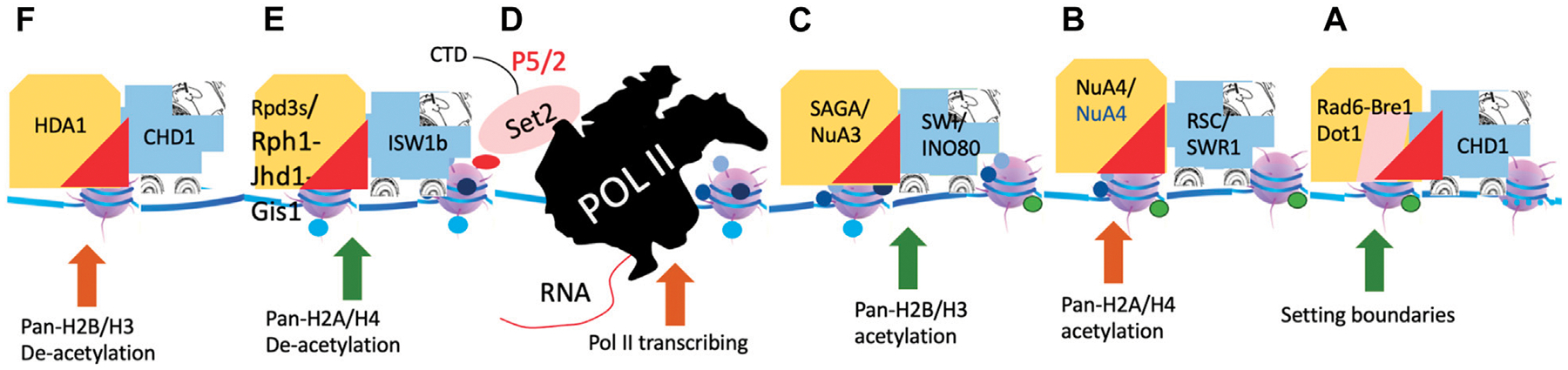
A hypothetical model of chromatin remodelers and histone modification complexes. **(A)**. The generation of H2Bub and H3K79me3 (not shown). **(B)**. The generation of Pan-H4ac and H2Aac. **(C)**. The generation of Pan-H3ac and H2BAac. **(D)**. Pol II transcription and the generation of H3K36me3. **(E)**. The removal of all acetylated groups on H2A and H4 and methyl groups on H3K36me3. **(F)**. The removal of acetylated groups on H2B and H3.

Transcription regulation in eukaryotes has long been a mystery in the transcription and epigenetic field. People try to use different codes to interpret the complexity of the process, however, but it does not seem to help much. There is an urgent need to integrate all the scattered information that has accumulated in the transcription and epigenetic fields over the last seven or 8 decades. Here, I present a simple and unified model for a general transcriptional regulatory mechanism that integrates a large group of transcriptional cofactors together to depict the intrinsic coupling of these cofactors and their cognate roles after a driver function of chromatin remodelers is assigned. It could be summarized in a simple cartoon, which I called “Great Truths Are Always Simple” (Figure 13).

## Data Availability

The original contributions presented in the study are included in the article/Supplementary Material, further inquiries can be directed to the corresponding author.
